# Effect of Coating Process on Mechanical, Optical, and Self-Healing Properties of Waterborne Coating on Basswood Surface with MF-Coated Shellac Core Microcapsule

**DOI:** 10.3390/polym13234228

**Published:** 2021-12-02

**Authors:** Yu Tao, Xiaoxing Yan, Yijuan Chang

**Affiliations:** 1 Co-Innovation Center of Efficient Processing and Utilization of Forest Resources, Nanjing Forestry University, Nanjing 210037, China; taoyu@njfu.edu.cn; 2 College of Furnishings and Industrial Design, Nanjing Forestry University, Nanjing 210037, China; changyijuan@njfu.edu.cn

**Keywords:** self-repairing microcapsules, coating process, waterborne coatings

## Abstract

Self-repairing microcapsules prepared with melamine formaldehyde (MF) resin as wall material and shellac and waterborne coating as core material were added to waterborne coating to prepare a self-repairing coating. In order to explore the effect of the coating process on the performance of the waterborne coating on the basswood surface with microcapsules, the number of coating layers of primer and finish and the addition mode of the microcapsules were tested as influencing factors. The effects of different coating processes on the optical, mechanical, and liquid resistance of the basswood surface coating were investigated. The results showed that different coating processes had little effect on the color difference of the coating. When the coating process was two layers of primer and three layers of finish, and microcapsules were added to the finish, the minimum gloss of the basswood surface coating at 60° incident angle was 10.2%, and the best mechanical properties, liquid resistance, and comprehensive properties were achieved. Finally, the aging resistance and self-healing performance of the waterborne coating on the basswood surface prepared by this coating process were explored. The results showed that the waterborne coating had a certain repair effect on scratch damage. This paper lays a theoretical foundation for the practical application of self-healing microcapsules in wood-surface waterborne coatings.

## 1. Introduction

As a green environmental-protection material, wood is widely used in furniture manufacturing and other industries [1,2]. However, because wood contains lignin, cellulose, and hemicellulose, it is easily degraded into glucose, water, and carbon dioxide, resulting in wood deterioration and shortened service life [3,4,5]. Therefore, the use of waterborne wood coatings can close wood pipe-holes, protect wood surface quality, and improve wood utilization [6,7]. However, waterborne wood coatings will inevitably be disturbed by the external environment in the process of use, and it is very easy to produce micro-cracks due to factors such as wet expansion and dry shrinkage of the wood substrate [8,9]. Self-repairing microcapsule technology has been introduced into the field of wood coatings [10,11,12], so as to achieve the purpose of repairing coating cracks and improve the usability and safety of wood coatings [13]. When cracks occur in the coating, the self-repairing waterborne coating can automatically repair the cracks [14]. This technology is of great significance in regulating the characteristics of wood surface coating and improving its defects.

Microcapsule technology has strong universality and has been integrated into material manufacturing, food production and other processes. The choice of core and wall materials has an important impact on the properties of microcapsules. Xiao et al. [15] prepared microcapsules with cellulose nano cellulose (CNF) as shell wall material and isophalon diisocyanate (IPDI) as crosslinking agent. The results showed that n-hexadecane microcapsules have adjustable release characteristics varying with temperature, and the microcapsule release system has great potential in the development of efficient and environmentally friendly pesticide preparations. Wang et al. [16] used capric acid (CA) as the core material and nano SiC-modified melamine urea formaldehyde resin (MUF) as the wall material to prepare phase-change material microcapsules with high photothermal conversion performance by ultrasonic dispersion in situ polymerization. The results showed that when the content of nano SiC reached 6wt%, the phase-change microcapsule with packaging efficiency of 65.7% had the best performance, and the thermal conductivity increased by 59.2%. When in situ polymerization is used, the selection of emulsifier and the determination of emulsifying speed are the key factors for the preparation of high-quality microcapsules. Emulsifier is very important for the stability of the overall structure of microcapsules. At the same time, finding the appropriate emulsifying speed is also an important and difficult point in the preparation of microcapsules [17]. Wei et al. [18] studied the influencing factors of emulsion stability, and used the new sieve-plate rotary emulsification device instead of the traditional high-speed shear emulsifier to prepare the emulsion, and compared the stability of the emulsion prepared by different methods, in order to better simulate the emulsification process of polymer surfactants in the course of formation. The results showed that the stability of polymer surfactant emulsion increased with the increase of polymer surfactant concentration, salt concentration, and water/oil ratio. Ma et al. [19] optimized the emulsification method and prepared probiotic microcapsules with milk protein as wall material. The results showed that the concentration of skimmed milk was 35%, the emulsification time was 10 min, and the stirring speed was 600 rpm. The microcapsule technology could be used to produce functional and healthy foods containing probiotics. Patel et al. [20] used jackfruit seed starch (JSS) and soybean protein isolate (SPI) as encapsulation material and NBRE-15 as emulsifier. The results showed that the storage of anthocyanin half-life in microcapsules at room temperature (37 °C) had higher stability. When microcapsules were prepared by in situ polymerization, urea formaldehyde resin was used as wall material, which would reduce the coating rate [21]. The existing microcapsules had the defects of poor thermal stability and long service life. In this paper, self-repairing microcapsules were prepared with melamine formaldehyde (MF) resin as wall material and shellac and waterborne coating as core material, and added to waterborne coating on the wood surface [22]. The performance of waterborne coating on the wood surface was not only affected by the content of the microcapsules added into the coating, but also by the coating process. This is an important factor that cannot be ignored [23]. In order to further determine the self-healing mechanism of waterborne coating, the coating times of primer and finish, and whether adding microcapsules in the primer or finish has an impact on the performance of the coating was examined.

Previous research focused on the preparation process of microcapsules [24]. Based on these previous research results, our research considered the coating process of the waterborne coating, and studied the effects of different coating processes on the self-healing performance, mechanical properties, and aging resistance of the coating with microcapsules. The prepared MF-resin-coated shellac and waterborne coating microcapsules were added to the waterborne coating in different ways, and the coating times of primer and finish in the coating process were changed. The optical, mechanical, and liquid resistance properties of the waterborne coating on the surface of the basswood were tested [25,26], its microstructure was observed, its chemical composition was analyzed, and the influence of the coating process of adding microcapsules on the performance of the waterborne coating was analyzed and summarized, so as to determine the best coating process for preparing self-healing waterborne coating. Then, the aging resistance and self-repairing performance of the basswood surface coating with the best coating process were tested [27,28], and the influence of the coating process on the self-repairing performance of waterborne coating on the wood surface was analyzed to prepare self-healing waterborne coatings with good repair performance. This laid a theoretical foundation for the application of self-healing microcapsules in wood-surface waterborne coating.

## 2. Materials and Methods

### 2.1. Experimental Materials

The basic introduction and production sources of the experimental materials are listed in Table 1. The main components of waterborne primer and finish included waterborne acrylic acid copolymer dispersion, matting agent, additive, and water, and the solid content is 30%.

### 2.2. Preparation of Microcapsules

The melamine:formaldehyde:water was mixed according to the mass ratio of 1:2:5, triethanolamine was dropped to adjust to weak alkalinity, and the wall material prepolymer was obtained by continuous stirring at 600 rpm in a 70 °C water bath for 30 min. The waterborne primer and 20% shellac ethanol solution were mixed and evenly stirred. The emulsifier was added into the mixture of shellac and waterborne primer. The core material emulsion was obtained by reacting for 60 min at a certain speed in a constant-temperature water bath of 70 °C. Finally, the wall material was slowly added to the core material emulsion. At the rotational speed of 600 rpm, the pH value was adjusted to 2.5–3.0 by citric acid, and the mixture was slowly heated to 70 °C for 3 h.

### 2.3. Technological Process of Coating

The coating-process experiment schedule is shown in Table 2. The primer and finish coating were applied in two and three layers, and the microcapsules were added in the primer and finish paint. The specific ingredients are shown in Table 3. According to previous research [24], when microcapsules prepared at 600 rpm were added to the coating with the concentration of 5.0%, the comprehensive performance of the coating was the best. Taking sample 1# as an example, the 0.2 g prepared microcapsules were added to 1.8 g primer, the primer was coated to the basswood surface with a brush and then pushed flat with a paint-film preparer. The coating was dried at room temperature for 10 min, and then put into a 35 °C blast-drying oven for 20 min. After the surface of the coating was dry, it was gently polished with 800-mesh sandpaper, then coated with the second primer and dried and polished repeatedly. The 2.0 g finish was weighed and prepared according to the brushing method of the primer. Experimental samples 2#–8# were prepared according to the above steps, and the thickness of the dry coating was about 60 μm.

### 2.4. Testing and Characterization

The instrument model, test type, and manufacturer used for the test are shown in Table 4. In terms of color value of the coating, L* is brightness, a* is red and green, b* is yellow and blue, c* is degree of color saturation, and H* is hue [29,30,31]. The color difference (ΔE*) of different samples can be calculated according to the formula:

ΔE* = [(ΔL*)^2^ + (Δa*)^2^ + (Δb*)^2^]^1/2^

The liquid-resistance experiments of coatings prepared by different coating processes were carried out at room temperature. The 15% NaCl solution, 70% medical ethanol, detergent, and red ink were selected as liquid-resistance reagents. The filter paper was soaked in NaCl solution, medical ethanol, detergent, and red ink, and then placed on the coating surface. The coating was then covered with a glass cover and left for 24 h (h). The chromaticity values before and after liquid resistance were recorded for color difference calculation, and the gloss of the measured position at 60° incident angle before and after liquid resistance were recorded.

The basswoods prepared with the two layers of primer and three layers of finish, with and without microcapsules added in the finish were placed in 100 °C and 150 °C electric blast-drying ovens for 48 h and then the ultraviolet weather-resistance tester for 240 h. The color value and gloss were measured every 8 h and every 40 h. The coating prepared by the best coating process and the coating prepared without microcapsules on two layers of primer and three layers of finish were compared by scratch-repair experiment. The coating was scratched with a blade with the same strength, and the self-repairing performance of the coating was observed every 7 days (d) with a ZEISS optical microscope (OM) AX10, which was produced by Carl Zeiss AG, Aalen, Germany. All the tests were repeated four times with the error less than 5%.

## 3. Results and Discussion

### 3.1. Micro and Chemical Composition Analysis of Microcapsules

**Figure 1:**
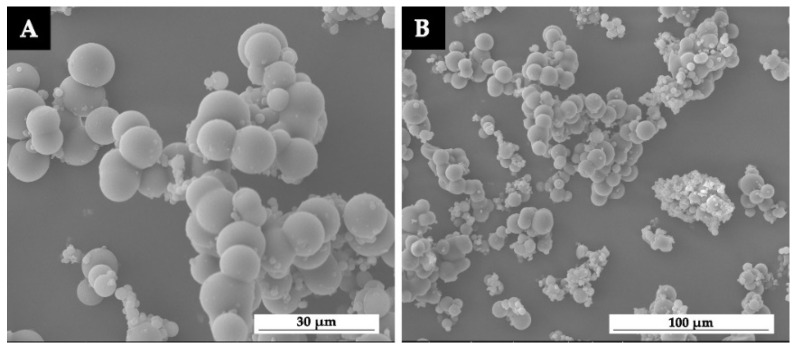
The SEM morphology of microcapsules prepared at 600 rpm: (**A**) high magnification and (**B**) low magnification.

**Figure 2:**
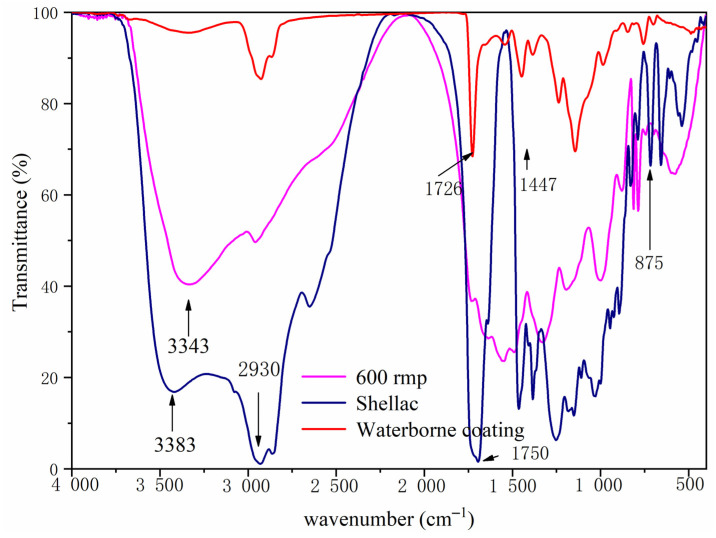
The FTIR of microcapsules and core materials.

The SEM and FTIR of microcapsules prepared at 600 rpm are shown in Figure 1 and Figure 2. Microcapsules were complete spheres with a smooth surface. Although there was a small amount of agglomeration, the particle size was basically uniform and the preparation was in good condition. The 1726 cm^−1^ represented the characteristic peak of C=O in waterborne acrylic resin, and the prepared microcapsules also had this peak, indicating that the core material waterborne primer was successfully coated. The stretching vibrations of -COOH and -CH_2_ groups in shellac were 1750 cm^−1^ and 2930 cm^−1^, respectively, and the corresponding peaks also appeared on the microcapsule curve, indicating that the core shellac was successfully coated and the microcapsules were successfully prepared.

### 3.2. Effect of Coating Process on Optical Properties of Waterborne Coatings with Microcapsules

When measuring the chromaticity value, two points on the same basswood paint film were taken. L* is brightness, a* is red and green, b* is yellow and blue, c* is degree of color saturation, and H* is hue. As can be seen from Table 5, the color difference (ΔE*) of sample 4# was the lowest, followed by sample 6#. The color difference (ΔE*) of samples 1–8# was very small, indicating that the coating process did not affect the color difference of coating on the basswood. The coating process of sample 4# was three layers of primer and three layers of finish, and the microcapsules were added with the primer.

The gloss values of the paint film on the basswood at 20°, 60°, and 85° incident angles were measured. It can be seen from Table 6 that the microcapsules of samples 1#–4# were added in the primer and the microcapsules of samples 5#–8# were added in the finish. At the incident angle of 60°, the gloss of samples 1#–4# was higher than that of samples 5#–8#, because the addition of microcapsules in the finish affected the transmittance of the finish. Sample 6# (two layers of primer, three layers of finish, and microcapsules added to the finish) had the lowest gloss. Sample 6# (three layers of primer, three layers of finish, and microcapsules added to the primer) had the highest gloss.

### 3.3. Effect of Coating Process on Mechanical Properties of Waterborne Coatings with Microcapsules

It can be seen from Table 7 that among the mechanical properties of samples 1#–8#, the mechanical properties of sample 6# (two layers of primer, three layers of finish, and microcapsules added to the finish) were the best. The hardness was 5H, the adhesion grade was grade 1, the impact resistance was 7 kg·cm, and the elongation at break was 16.28%.

### 3.4. Effect of Coating Process on Liquid Resistance of Basswood Waterborne Coating with Microcapsules

The color difference (ΔE*) of the coatings on the basswood before and after liquid resistance prepared by different coating processes is shown in Table 8. Among them, the color difference values of sample 6# before and after liquid resistance to NaCl, ethanol, and detergent were relatively small; in particular, the color difference value of red ink was the smallest among the samples 1#–8#. The gloss results of the coating after liquid resistance at an incident angle of 60° are shown in Table 9. This was lower than that of most samples before liquid resistance in Table 6. The gloss of sample 6# changed little before and after liquid resistance.

The classification of liquid resistance of paint films is shown in Table 10. The influence of the coating process on the liquid-resistance grade of basswood paint film with microcapsules is shown in Table 11. The waterborne coating on the surface of the basswood had the same liquid-resistance grade to NaCl, ethanol, and detergent, which was grade 1, and the liquid-resistance grade to red ink was mostly grade 2. Because microcapsules are powdery substances, it was easier to adsorb red ink by adding microcapsules in the finish than in the primer. The liquid-resistance grade of sample 6# and sample 8# was grade 1, indicating that sample 6# (two layers of primer, three layers of finish, and microcapsules added to the finish) had better liquid resistance.

### 3.5. Microstructure and Chemical Composition Analysis of Coating on Basswood Surface

**Figure 3:**
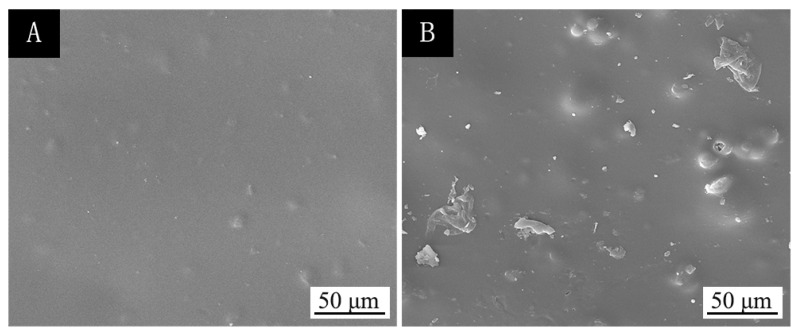
SEM images of two bottom and three surface coatings with different microcapsule concentrations: (**A**) 0 and (**B**) 5.0%.

Considering the influence of the coating process on the optical properties, mechanical properties, and liquid resistance of basswood paint film, the performance of the surface coatings was the best when the coating process was two layers of primer and three layers of finish and microcapsules were added to the finish. The SEM images of two layers of primer and three layers of finish without microcapsules and with 5.0% microcapsules are shown in Figure 3. Compared with the coating without microcapsules, the surface of the paint film with 5.0% microcapsules was more granular. Considering the repair effect of microcapsules, adding microcapsules in the finish can give a better repair effect.

**Figure 4:**
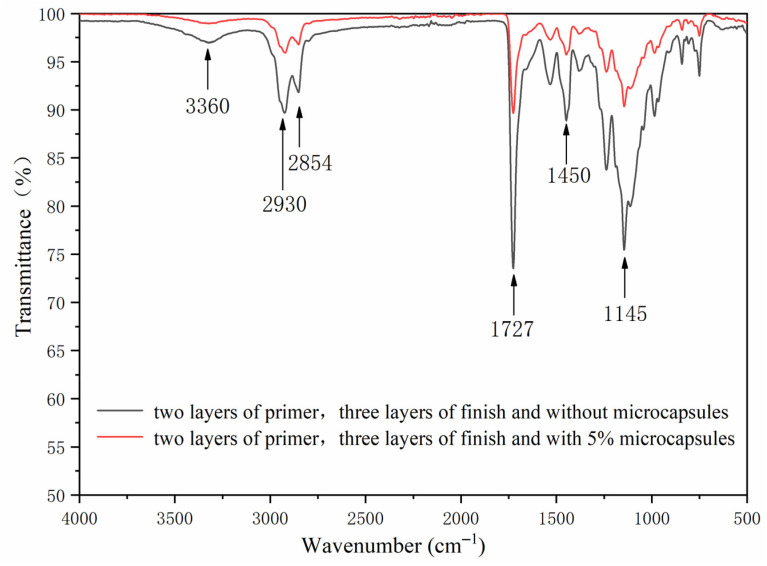
The FTIR of basswood surface coating with three layers of primer and two layers of finish with different microcapsule concentrations.

The FTIR of the basswood waterborne coating with two layers of primer, three layers of finish without microcapsules, and 5.0% microcapsules are shown in Figure 4. The stretching vibration absorption peaks of 2930 cm^−1^ and 1450 cm^−1^ in MF resin were –CH_2_ and C=N bonds, respectively. The 1726 cm^−1^ represented the characteristic peak of C=O in microcapsule-waterborne coating, and 3360 cm^−1^ is the N–H absorption peak in MF resin, indicating that the waterborne coating and shellac are coated successfully. In the coating process with microcapsules, the peak value of the basswood paint film became shorter, and the peak trends of basswood paint film without microcapsules and with microcapsules were basically the same. As the core material of microcapsules contained waterborne coatings, the composition of the basswood paint film changed little.

### 3.6. Effect of Microcapsule Content on Aging Resistance of Basswood Surface Coating

**Figure 5:**
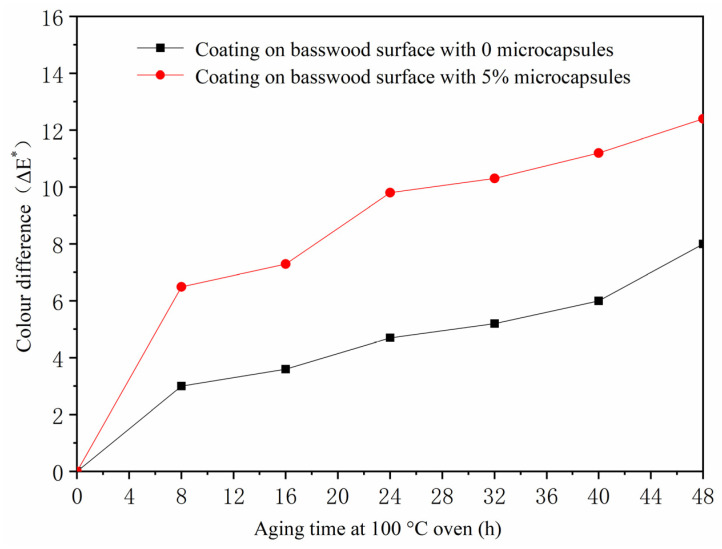
The influence of oven aging time at 100 °C on color difference of basswood paint film.

**Figure 6:**
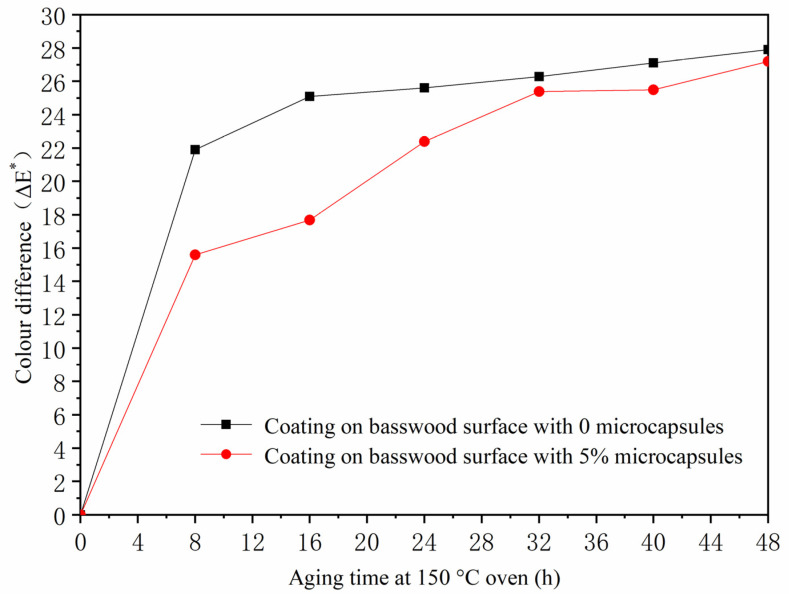
The influence of oven aging time at 150 °C on color difference of basswood coating.

According to the above experiments, it was found that the coating process of the two-layer primer and the three-layer finish, the coating performance was better when microcapsules were added to the finish. Therefore, the two-layer primer and three-layer finish did not add microcapsules, and the coating on the surface of the basswood board with the concentration of 5.0% microcapsules was added for the aging test. The influence of aging time on the change of paint film color difference is shown in Table 12. L* is brightness, a* is red and green, b* is yellow and blue, c* is degree of color saturation, and H* is hue. The a* value in Table 11 represents the red-green value. Shellac is purple-red. In the 100 °C aging experiment (Table 12, Figure 5), the a* value of the coating without adding microcapsules changed from 19.3 to 12.8, a decrease of 6.5, and the color difference increased from 3.0 to 8.0, indicating that the coating was aging after being heated for a long time. The a* value of the coating with 5.0% microcapsules changed from 17.5 to 13.6, which was smaller than the decrease range of the a* value without adding microcapsules. At the same time, the color difference value increased from 6.5 to 12.4. Adding microcapsules during the aging experiment at 100 °C produced a greater color difference than without adding microcapsules, and the temperature at this time was not enough to carbonize the wood. In the 150 °C aging experiment (Table 12, Figure 6), the a* value of the coating without adding microcapsules changed from 14.2 to 18.0, an increase of 3.8. At this time, the basswood was carbonized due to the high temperature of the oven. After long-term heating, the coating aged, and the color difference increased from 21.9 to 27.9. The a* value of the coating with 5% microcapsules increased from 15.1 to 19.4, and the color difference increased from 15.6 to 27.2. Compared with no microcapsules, increase in the range of the a* value of the coating with microcapsules was smaller. It can be seen that the addition of microcapsules made the red-green color of the coating more stable.

**Figure 7:**
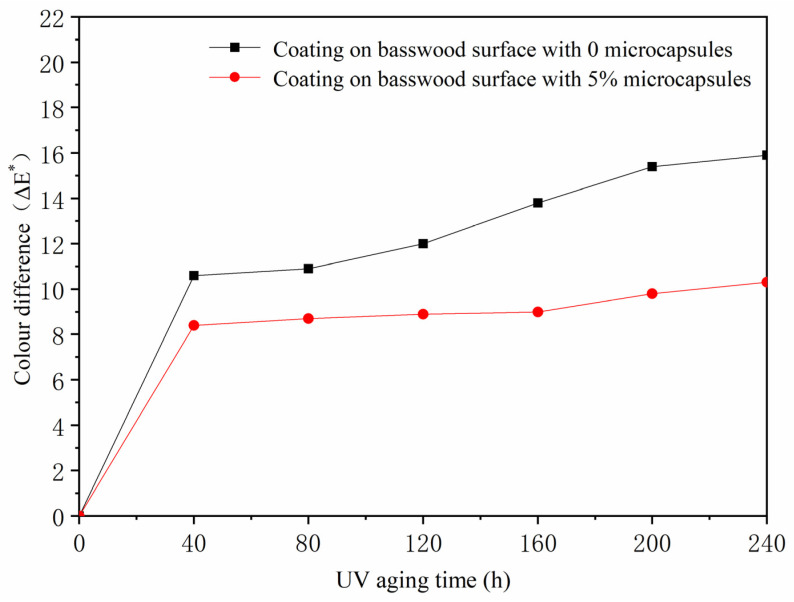
The influence of UV aging time on color difference of basswood coating.

Ultraviolet-light aging had a significant influence on the color of shellac. The a* value changed irregularly (Table 12). The color difference of the coating without microcapsules was larger than that with microcapsules after the ultraviolet-light aging experiment (Figure 7). The reason was that after ultraviolet light was irradiated, the surface of the coating had micro-cracks, the microcapsules were broken, and the mixed solution of shellac and waterborne coating flowed out. The ultraviolet light affected the color of shellac, and the repair agent repaired the paint film. The micro-cracks improved the performance of the basswood surface coating with microcapsules.

**Figure 8:**
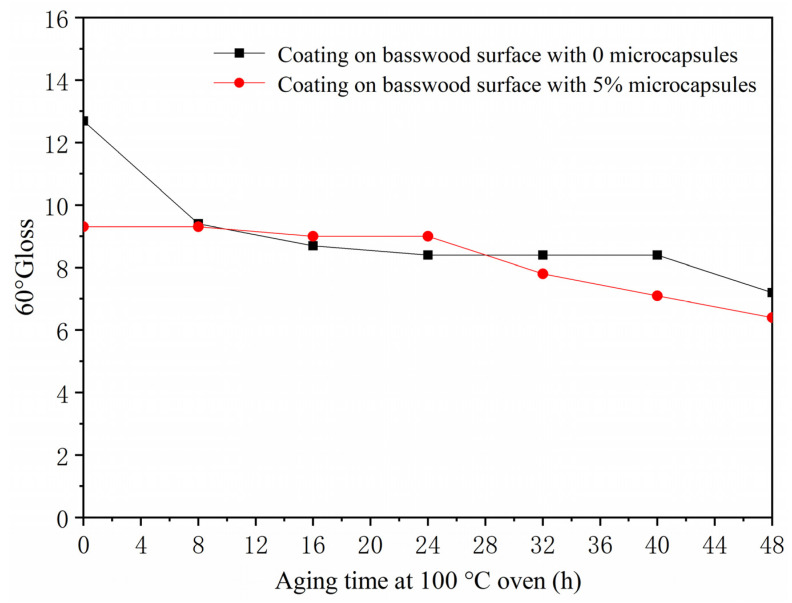
The influence of oven aging time at 100 °C on gloss of basswood film.

**Figure 9:**
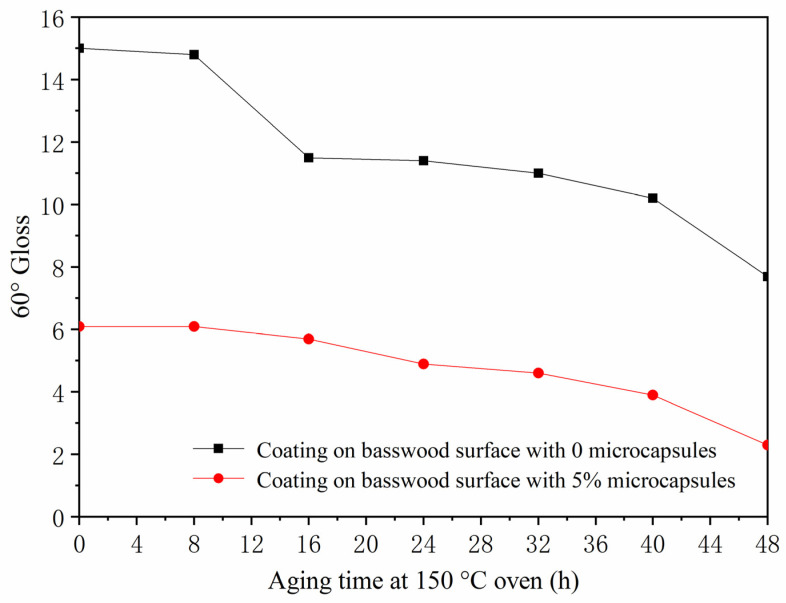
The influence of oven aging time at 150 °C on gloss of basswood film.

**Figure 10:**
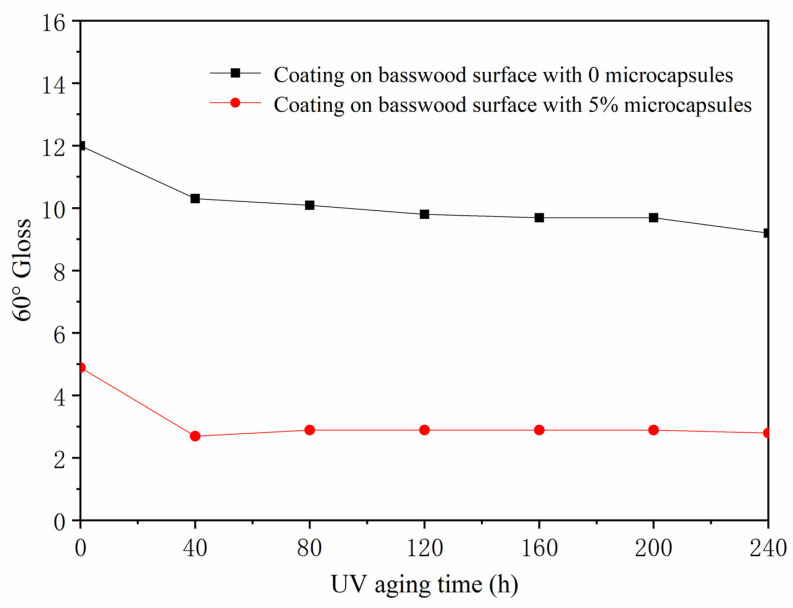
The influence of UV aging time on gloss of basswood film.

The gloss values of the surface coating of basswood after artificial accelerated aging at three incident angles (20°, 60°, and 85°) are shown in Table 13. The gloss value of the paint film at an incident angle of 60° was plotted for analysis. When the coating on the surface of basswood was aged in a 100 °C oven for 48 h (Figure 8), the gloss of the coating without microcapsules and with 5.0% microcapsules both showed a downward trend. The gloss of the coating without microcapsules decreased from 12.7 to 7.2, and the gloss of the coating with 5.0% microcapsules dropped from 9.3 to 6.4. The gloss of the coating with microcapsules decreased more slowly than the coating without microcapsules. When the coating on the surface of basswood was aged for 48 h in a 150 °C oven (Figure 9), the gloss of the coating without and with 5.0% microcapsules also decreased, the degree of gloss of the coating without microcapsules dropped from 15.0 to 7.7, the gloss of the coating with 5.0% microcapsules dropped from 6.1 to 4.9, and the decline in gloss was also slower than the coating without microcapsules. After 240 h of UV aging (Figure 10), the surface gloss of the coating with 5.0% microcapsules decreased. The first 40 h showed a downward trend. From 40 to 240 h, the gloss of the coating on the surface of the wood was almost unchanged, and the gloss was relatively stable. It can be seen that the coating on the surface of basswood with microcapsules was more stable in aging resistance and had higher gloss than the coating without microcapsules.

**Figure 11:**
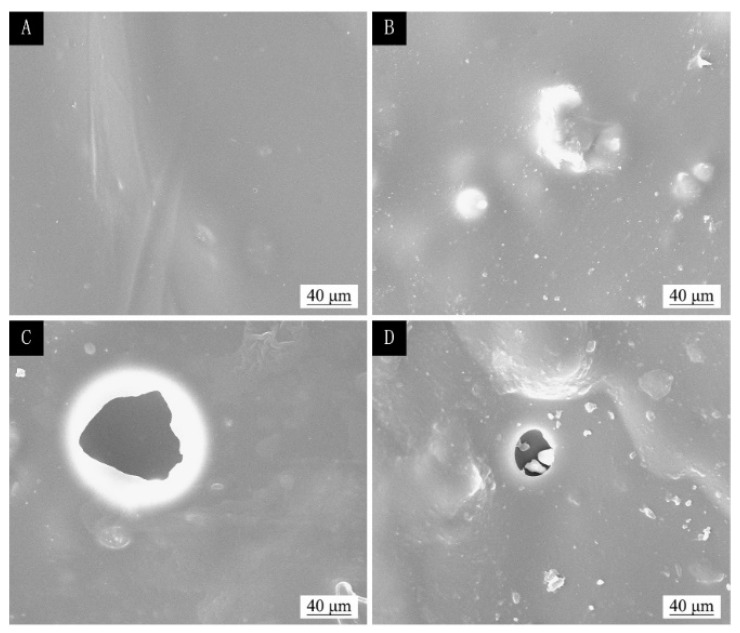
The SEM of basswood film with different microcapsule concentrations before and after 100 °C aging: before aging, (**A**) 0% and (**B**) 5.0%; after aging, (**C**) 0%, and (**D**) 5.0%.

**Figure 12:**
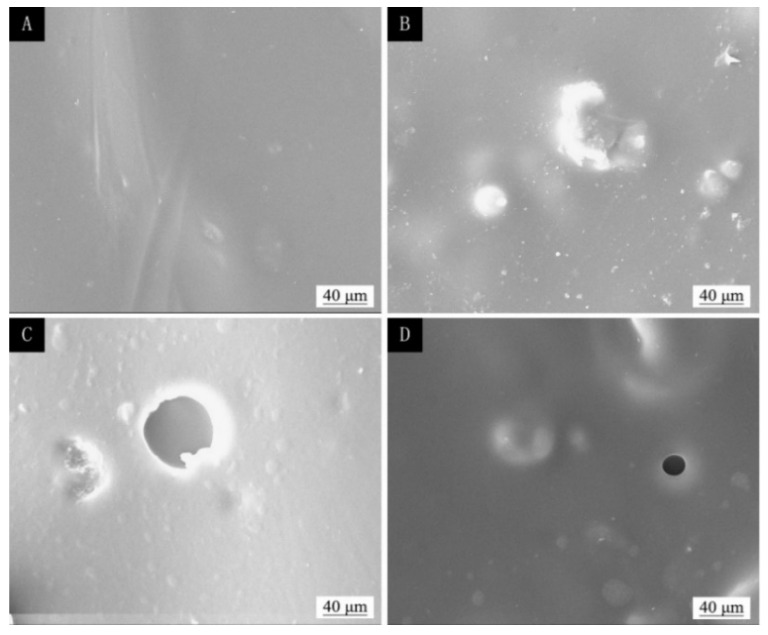
The SEM of basswood film with different microcapsule concentrations before and after 150 °C aging: before aging, (**A**) 0% and (**B**) 5.0%; after aging, (**C**) 0%, and (**D**) 5.0%.

**Figure 13:**
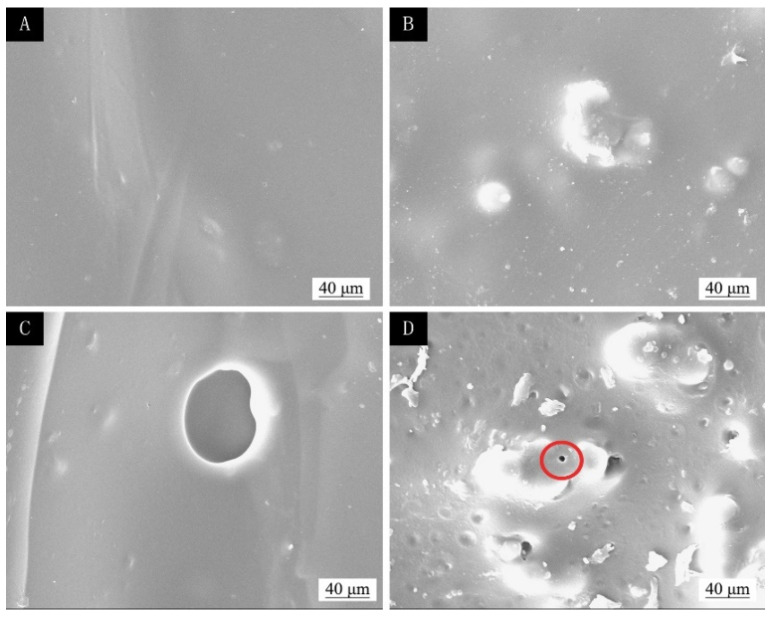
The SEM of basswood film with different microcapsule concentrations before and after UV aging: before aging, (**A**) 0% and (**B**) 5.0%; after aging, (**C**) 0%, and (**D**) 5.0%.

The SEM of the coating before and after temperature aging and ultraviolet light aging are shown in Figure 11, Figure 12 and Figure 13. Figure 11A, Figure 12A and Figure 13A show the coating of two-layer primer and three-layer paint without adding microcapsules before aging, and their surfaces were relatively smooth. Figure 11B, Figure 12B and Figure 13B show the coating of the two-layer primer and three-layer paint with 5.0% microcapsules added before aging. Because the microcapsules were added in the finish, the surface of the coating was not smooth and had a grainy feel. Figure 11C shows the morphology of the coating without adding microcapsules after being aged in an oven at 100 °C; large irregular-shaped cracks can be seen. Figure 11D shows the paint film after adding 5.0% microcapsules and passing it through the oven at 100 °C. Figure 12C shows the morphology of the coating without adding microcapsules after being aged in an oven at 150 °C; large irregular-shaped cracks can be seen. Figure 12D shows the linden-wood paint film after adding 5.0% microcapsules and passing it through the oven. The morphology after aging at 150 °C also produced small round cracks. Figure 13C shows the morphology of the coating without adding microcapsules after aging with UV light, and broad bean-shaped cracks were produced. Figure 13D shows the morphology of the coating with adding 5.0% microcapsules after UV aging. The morphology had created smaller circular cracks. The cracks of the coating with microcapsules were relatively small after aging. The reason was that the microcapsules had a specific adhesion effect on the stability of the paint film, which could improve the quality of the paint film.

**Figure 14:**
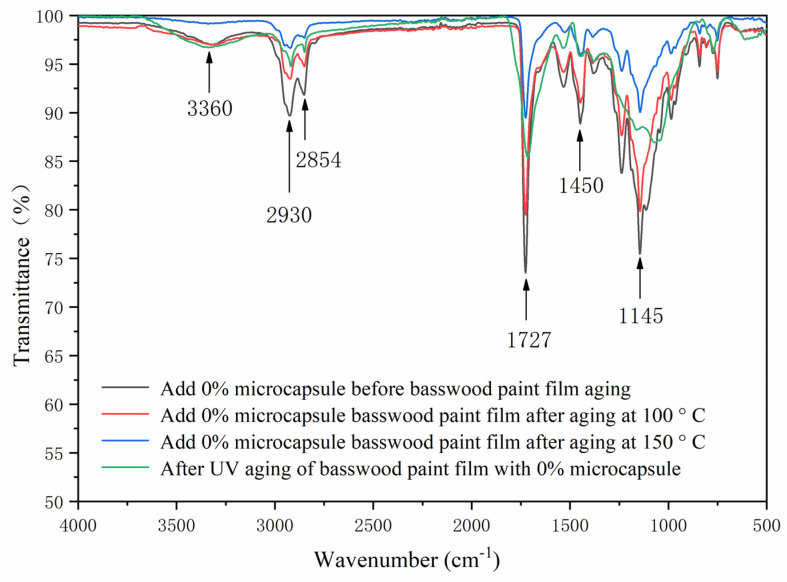
The FTIR of basswood film without microcapsules before and after aging.

**Figure 15:**
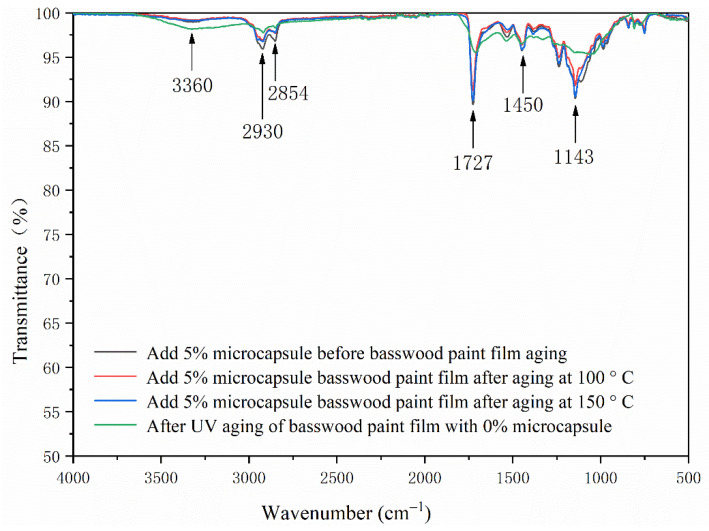
The FTIR of basswood film with 5.0% microcapsules before and after aging.

The FTIR of coating on basswood surface before and after the aging is shown in Figure 14 and Figure 15. The C–H bending vibration absorption peaks at around 1450 cm^−1^, 2930 cm^−1^ and 2854 cm^−1^ were the characteristic absorption peaks of –CH_2_ stretching vibration, and 1727 cm^−1^ was the characteristic peak of C=O in waterborne coating. The N–H absorption peak in the wall material was 3360 cm^−1^. After aging, the peak of coating on the basswood surface became shorter, especially after UV aging. However, no peak disappeared or appeared after aging, indicating that the coating on the basswood surface without and with 5.0% microcapsules was artificially accelerated. The composition had not changed before or after. The chemical composition results showed that after artificially accelerated aging experiments, different aging conditions would not change the composition of the coating on the basswood surface without or with the addition of 5.0% microcapsules.

### 3.7. Effect of Microcapsule Concentration on the Self-Repairing Performance of Coating

**Figure 16:**
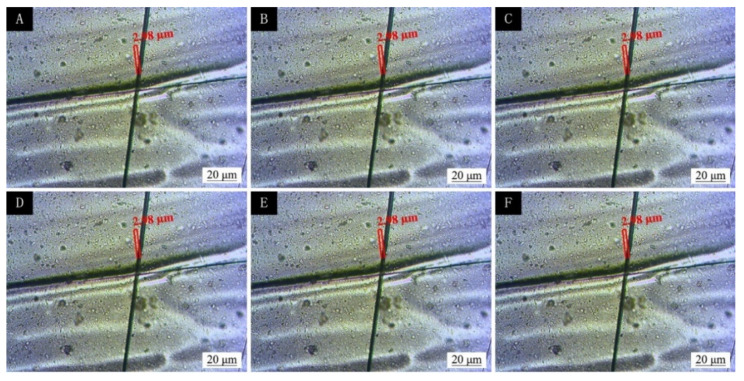
Scratch days for two layers of primer and three layers of finish without microcapsules added to the finish coating (**A**) 1 d, (**B**) 3 d, (**C**) 5 d, (**D**) 7 d, (**E**) 9 d, and (**F**) 11 d.

**Figure 17:**
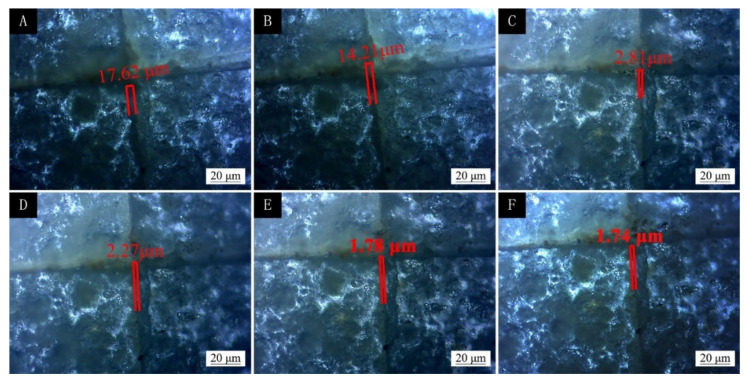
Scratch days for two layers of primer and three layers of finish with 5% microcapsules added to the finish coating (**A**) 1 d, (**B**) 3 d, (**C**) 5 d, (**D**) 7 d, (**E**) 9 d, and (**F**) 11 d.

**Figure 18:**
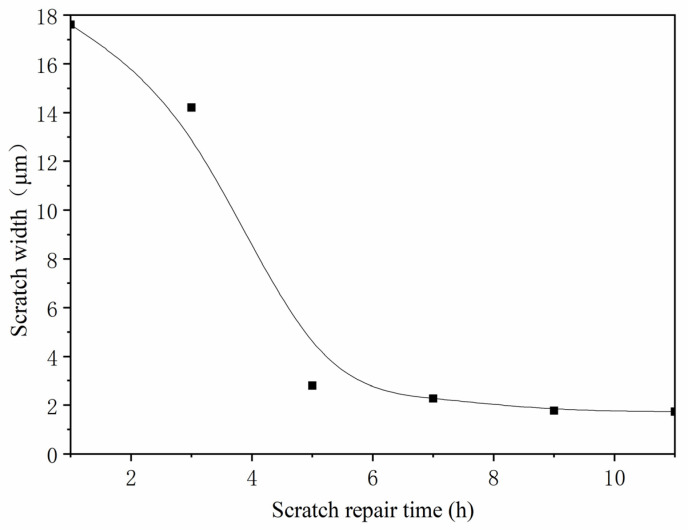
Scratch repair curve with time.

The coating was prepared by the best coating process (two-layer primer, three-layer paint, and 5.0% microcapsules added to the finish) and a two-layer primer and three-layer paint without microcapsules. The different kinds of coating prepared were scratched for comparison. From the 1st day to the 11th day, the observations were made every two days. The observation results are shown in Figure 16 and Figure 17. It can be seen from Figure 16 that the scratches on the coating without microcapsules did not become smaller after 11 d, while the scratches on the coating with microcapsules changed from the initial 17.62 μm to 1.74 μm, and the scratches were reduced, nearly ten times, so it can be seen that the microcapsules had a certain repair effect. The curve of scratch repair over time is shown in Figure 18. The scratch repair efficiency was highest in the first 5 d, from 17.62 μm to 2.81 μm, and the width of the scratch was reduced by nearly nine times. The scratch repair efficiency slowed down from the 7th day, from 2.27 μm to 1.74 μm, and the scratches were reduced by less than two times. When cracks appeared on the surface of the coating, the microcapsules broke and the mixed solution of shellac and waterborne coating flowed out, which repaired the micro-cracks of the coating and improved the performance of the microcapsule basswood paint film. With the extension of the repair time, the shellac and waterborne-coating mixed solution had gradually cured, and the repair effect was weakened after 7 d. The repair mechanism of polymers was the intrinsic self-repair, which was driven by macromolecular interactions. The repair effect was enhanced when stimulated by external triggers, such as thermal, photochemical, and electrical effects.

## 4. Conclusions

The experiments showed that the coating process does not affect the color difference of the coating on the surface of basswood. When the coating process was the two-layer primer, three layers of finish, and microcapsules added to the finish, the gloss of the coating on the basswood surface was the lowest. At this time, the hardness of the coating was 5H, the adhesion level was 1, the impact resistance was 7 kg·cm, and the elongation at break was 16.28%. Among samples 1#–8#, the mechanical properties of the samples prepared with the coating process (two coats of primer and three coats of topcoat) were the best. The liquid-resistance grade of the coating on the basswood surface to NaCl, ethanol, and detergent was 1, and the liquid-resistance grade to red ink was 2. The samples prepared by the coating process (two-layer primer, three-layer finish, and microcapsules added in the finish coating) had better liquid resistance. In the 100 °C-temperature aging experiment, the color difference of the coating with microcapsules was larger than that without microcapsules, while in the 150 °C aging experiment, the increased range of a* value of the coating on the basswood surface with microcapsules was smaller than that without microcapsules. It can be seen that adding microcapsules could make the red and green of the coating more stable. The color difference of the coating with microcapsules was smaller than that without microcapsules after the temperature-aging test. In the scratch-repair experiment, the repair effect of the basswood paint film with microcapsules was obvious, and the repair effect reached the best in the first 7 d. This laid a theoretical foundation for the preparation of self-healing waterborne coatings with good repair performance and the application of self-healing microcapsules in waterborne coatings on wood surfaces.

## Figures and Tables

**Table 1:** Basic materials.

Materials	CAS	Molecular Mass (g/mol)	Source
Melamine	108-78-1	126.15	Henan Ningjie Chemical Products Co., Ltd., Henan, China
37.0% Formaldehyde	50-00-0	30.03	Jining Hengtai Chemical Co., Ltd., Jining, China
Citric acid	5949-29-1	210.14	Nanjing Chemical Reagent Co., Ltd., Nanjing, China
Triethanolamine	102-71-6	149.19	Henan Yuheng Chemical Co., Ltd., Henan, China
Basswood (65 mm × 65 mm × 4 mm)	-	-	Guangdong Yihua Life Science and Technology Co., Ltd., Guangdong, China
Waterborne coating (waterborne primer and finish)	-	-	Dulux Coatings (Shanghai) Co., Ltd., Shanghai, China
Shellac film			Jinan Dahui Chemical Technology Co., Ltd., Jinan, China
15.0% NaCl solution	-	-	Self-prepared solution at laboratory room temperature
Detergent	-	-	Shanghai and Huangbai cat Co., Ltd., Shanghai, China
70% Medical ethanol	-	-	Qingdao Heinrich Innoway Disinfection Technology Co., Ltd., Qingdao, China
Red ink	-	-	Shanghai Fine Cultural Products Co., Ltd., Shanghai, China

**Table 2:** Coating-process experiment schedule.

Samples	Primer Layers	Finish Layers	Addition Method of Microcapsules
1#	2	2	Primer
2#	2	3	Primer
3#	3	2	Primer
4#	3	3	Primer
5#	2	2	Finish
6#	2	3	Finish
7#	3	2	Finish
8#	3	3	Finish

**Table 3:** Coating process ingredients list.

Samples	Microcapsule (g)	Waterborne Primer (g)	Waterborne Finish (g)	Self-Repairing Waterborne Coating (g)
1#–4#	0.2	1.8	2.0	4.0
5#–8#	0.2	2.0	1.8	4.0

**Table 4:** Testing and characterization schedule.

Testing Type	Instrument	Model	Manufacturer
Color value of coating	Colorimeter	SEGT-J	Shaoxing Shangyu Aike Instrument Co., Ltd., Shaoxing, China
Gloss of coating	Glossmeter	HG268	Hangzhou Caipu Technology Co., Ltd., Hangzhou, China
Hardness of coating	Pencil-hardness tester	-	Beijing Airuipu Technology Co., Ltd., Beijing, China
Adhesion of coating	Film scribbler	QFH-HG600	Shanghai Qianshi Precision Electromechanical Technology Co., Ltd., Shanghai, China
Impact-resistance of coating	Film impactor	QCJ	Jinan Languang Electromechanical Technology Co., Ltd., Jinan, China.
Tensile strength of the coating	Precision electronic universal testing machine	AG-IC100KN	Shimadzu Co., Ltd., Kyoto, Japan
Aging resistance	Ultraviolet weather-resistance tester	ZN	Guangdong Hongkuo Test Equipment Co., Ltd., Dongguan, China
Micro morphology	Scanning electron microscope (SEM)	Quanta 200	FEI Company, Hillsboro, OR, USA
Chemical composition	Fourier transform infrared spectrometer (FTIR)	VERTEX 80V	Germany Bruker Co., Ltd., Karlsruhe, Germany

**Table 5:** Effect of coating process on color difference of microencapsulated basswood film.

Samples	L*_1_	a*_1_	b*_1_	c*_1_	H*_1_	L*_2_	a*_2_	b*_2_	c*_2_	H*_2_	ΔL*	Δa*	Δb*	ΔE*
1#	76.3	9.4	29.1	30.6	72.0	74.6	11.2	28.8	30.9	38.6	−1.7	1.8	−0.3	2.49
2#	65.7	8.3	28.9	30.1	73.9	65.7	13.1	27.9	30.1	73.9	0	4.8	−1.0	4.90
3#	76.8	10.5	26.2	28.2	68.0	75.2	13.1	26.5	29.5	63.6	−1.6	2.6	0.3	3.07
4#	67.5	13.2	27.0	30.1	63.8	67.2	13.9	27.0	30.4	62.7	−0.3	0.7	0	0.76
5#	75.1	10.5	28.9	30.8	69.9	75.4	11.5	31.0	33.1	69.6	0.3	1.0	2.1	2.35
6#	72.2	12.0	26.6	29.2	65.6	71.2	11.6	26.8	29.2	66.5	−1.0	−0.4	0.2	1.10
7#	64.9	16.5	27.6	32.2	59.1	65.8	16.7	28.6	32.2	59.1	0.9	0.2	1.0	1.36
8#	71.2	12.2	27.1	29.7	65.7	71.2	10.8	28.7	30.7	69.3	0	−1.4	1.6	2.13

**Table 6:** Effect of coating process on gloss of microencapsulated basswood film.

Samples	20° Gloss (%)	60° Gloss (%)	85° Gloss (%)
1#	5.0	19.9	30.0
2#	4.5	19.1	38.3
3#	3.5	13.0	20.6
4#	5.3	23.3	36.7
5#	5.3	18.1	12.3
6#	3.0	10.2	4.6
7#	5.6	20.2	15.3
8#	3.2	11.6	7.5

**Table 7:** The influence of the coating process on mechanical properties of microencapsulated basswood film.

Samples	Hardness	Adhesion (Grade)	Impact Resistance (kg·cm)	Elongation at Break (%)
1#	4H	0	4	11.28
2#	4H	0	6	15.91
3#	3H	1	4	6.47
4#	3H	0	4	12.81
5#	2H	0	4	12.50
6#	5H	1	7	16.28
7#	4H	1	4	10.38
8#	4H	1	7	12.41

**Table 8:** The influence of coating process on liquid color difference resistance of basswood coatings with microcapsules.

Samples	Color Difference (ΔE*) before and after Liquid Resistance			
	NaCl	Ethanol	Detergent	Red Ink
1#	9.1	17.5	8.7	56.6
2#	25.6	23.1	55.3	47.1
3#	11.5	12.0	7.0	31.2
4#	6.3	5.5	6.5	19.4
5#	9.4	13.5	14.2	45.8
6#	3.5	15.7	3.5	7.5
7#	1.7	44.2	1.7	5.4
8#	7.6	19.7	7.6	39.6

**Table 9:** The gloss of basswood surface paint film prepared by different coating processes at 60° incident angle after liquid resistance.

Samples	Gloss (%) after Liquid Resistance			
	NaCl	Ethanol	Detergent	Red Ink
1#	10.2	8.0	9.6	9.5
2#	8.5	9.4	8.6	10.0
3#	6.4	6.0	9.1	10.4
4#	8.9	10.0	10.9	10.6
5#	6.2	7.3	7.0	7.4
6#	10.3	10.3	10.2	10.4
7#	9.0	6.2	9.1	8.1
8#	10.2	8.0	9.6	9.5

**Table 10:** Classification of liquid resistance of paint film.

Grade	Change of Paint Film
Grade 1	No mark
Grade 2	Slight discoloration marks
Grade 3	Slight discoloration or obvious discoloration marks
Grade 4	Obvious changes, blisters, wrinkles, etc

**Table 11:** The influence of coating process on the liquid-resistance grade of microcapsule basswood paint film.

Samples	NaCl	Ethanol	Detergent	Red Ink
1#	1	1	1	2
2#	1	1	1	2
3#	1	1	1	2
4#	1	1	1	2
5#	1	1	1	2
6#	1	1	1	1
7#	1	1	1	2
8#	1	1	1	1

**Table 12:** The influence of aging time on color difference of basswood coating.

Aging Environment	Microcapsule Concentration (%)	Aging Time (h)	L*	a*	b*	c*	H*	ΔL*	Δa*	Δb*	ΔE*
100 °C Oven	0	0	61.1	19.3	31.7	37.1	58.6	−	−	−	−
		8	61.8	16.5	30.8	34.9	61.7	0.7	−2.8	−0.9	3.0
		16	60.2	16.8	29.2	33.7	60.0	−0.9	−2.5	−2.5	3.6
		24	62.1	14.7	31.9	35.2	65.2	1.0	−4.6	0.2	4.7
		32	61.8	15.2	28.6	32.5	61.9	0.7	−4.1	−3.1	5.2
		40	63.0	13.6	31.5	34.3	66.6	1.9	−5.7	−0.2	6.0
		48	65.1	12.8	34.1	36.4	69.3	4.0	−6.5	2.4	8.0
	5.0	0	54.7	17.5	23.7	29.5	53.4	−	−	−	−
		8	60.0	14.5	26.1	29.8	60.8	5.3	−3.0	2.4	6.5
		16	60.4	14.3	27.0	30.5	62.1	5.7	−3.2	3.3	7.3
		24	62.7	15.3	29.0	32.9	62.1	8.0	−2.2	5.3	9.8
		32	63.2	15.4	29.2	33.0	62.0	8.5	−2.1	5.5	10.3
		40	62.5	14.6	31.2	34.5	64.9	7.8	−2.9	7.5	11.2
		48	65.7	13.6	28.0	31.2	64.0	11.0	−3.9	4.3	12.4
150 °C Oven	0	0	56.8	14.2	18.6	23.4	52.6	−	−	−	−
		8	67.7	13.7	37.6	40.0	69.9	10.9	−0.5	19.0	21.9
		16	67.8	12.0	41.1	42.8	73.6	11.0	−2.2	22.5	25.1
		24	60.3	16.9	43.8	47.0	68.9	3.5	2.7	25.2	25.6
		32	50.7	19.6	43.6	47.8	65.7	−6.1	5.4	25.0	26.3
		40	57.3	18.2	45.4	48.9	68.1	0.5	4.0	26.8	27.1
		48	54.2	18.0	46.1	49.5	68.6	−2.6	3.8	27.5	27.9
	5.0	0	61.7	15.1	19.7	24.9	52.5	−	−	−	−
		8	65.1	9.7	33.9	35.3	73.9	3.4	−5.4	14.2	15.6
		16	49.7	12.4	32.4	34.7	68.9	−12.0	−2.7	12.7	17.7
		24	66.8	13.4	41.4	43.6	72.0	5.1	−1.7	21.7	22.4
		32	52.5	20.9	42.6	47.5	63.8	−9.2	5.8	22.9	25.4
		40	53.1	21.3	42.9	47.9	63.5	−8.6	6.2	23.2	25.5
		48	56.5	19.4	46.0	49.9	67.0	−5.2	4.3	26.3	27.2
Ultraviolet weather-resistance test chamber	0	0	67.0	9.9	25.5	27.4	68.7	−	−	−	−
		40	75.4	10.2	32.0	33.6	72.3	8.4	0.3	6.5	10.6
		80	75.9	10.0	31.8	33.3	72.5	8.9	0.1	6.3	10.9
		120	76.1	11.1	33.2	35.0	71.5	9.1	1.2	7.7	12.0
		160	72.7	12.0	37.9	39.8	72.4	5.7	2.1	12.4	13.8
		200	61.6	14.5	11.8	18.7	39.2	−5.4	4.6	−13.7	15.4
		240	77.2	9.3	37.7	38.9	76.1	10.2	−0.6	12.2	15.9
	5.0	0	63.3	14.2	23.7	27.6	59.0	−	−	−	−
		40	69.3	11.5	29.0	31.2	68.2	6.0	−2.7	5.3	8.4
		80	64.5	13.4	32.3	34.9	67.3	1.2	−0.8	8.6	8.7
		120	69.0	10.6	29.5	31.4	70.1	5.7	−3.6	5.8	8.9
		160	67.9	12.5	31.3	33.7	68.2	4.6	−1.7	7.6	9.0
		200	68.9	11.8	31.4	33.6	69.4	5.6	−2.4	7.7	9.8
		240	71.0	10.1	29.2	30.9	70.9	7.7	−4.1	5.5	10.3

**Table 13:** The influence of aging time on color difference of basswood coating.

Aging Environment	Microcapsule Concentration (%)	Aging Time (h)	20° Gloss (%)	60° Gloss (%)	85° Gloss (%)
100 °C Oven	0	0	2.7	12.7	22.6
		8	1.8	9.4	21.8
		16	2.0	8.7	12.1
		24	2.1	8.4	11.9
		32	1.9	8.4	11.8
		40	1.9	8.4	21.7
		48	1.6	7.2	11.2
	5.0	0	2.4	9.3	4.2
		8	2.3	9.3	3.8
		16	2.2	9.0	3.6
		24	2.2	9.0	3.8
		32	2.0	7.8	3.4
		40	1.9	7.1	3.1
		48	2.1	6.4	1.5
150 °C Oven	0	0	3.2	15.0	31.6
		8	3.3	14.8	30.6
		16	3.0	11.5	19.3
		24	2.4	11.4	21.7
		32	2.1	11.0	23.3
		40	2.0	10.2	23.6
		48	1.7	7.7	29.0
	5.0	0	1.9	6.1	2.7
		8	1.9	6.1	2.7
		16	1.6	5.7	3.4
		24	1.5	4.6	2.3
		32	1.0	3.9	2.0
		40	0.9	2.3	0.0
		48	1.2	4.9	3.4
Ultraviolet weather-resistance test chamber	0	0	3.1	12.0	22.8
		40	2.7	10.3	23.1
		80	2.6	10.1	24.0
		120	2.7	9.8	18.5
		160	2.6	9.7	16.6
		200	2.6	9.7	16.6
		240	2.4	9.2	19.4
	5.0	0	1.6	4.9	2.4
		40	1.1	2.7	0.4
		80	1.2	2.9	0.5
		120	1.3	2.9	0.4
		160	1.3	2.9	0.4
		200	1.4	2.9	0.4
		240	1.2	2.8	0.4

## Data Availability

Not applicable.

## References

[1] Kumar A., Stanek K., Ryparova P., Hajek P., Tywoniak J. (2016). Hydrophobic treatment of wood fibrous thermal insulator by octadecyltrichlorosilane and its influence on hygric properties and resistance against moulds. Compos. Part B Eng..

[2] Sun Y., Wang C., Wu Y., Zuo J., Zhan X. (2020). Effect of nano-boron carbide on the properties of waterborne polyurethane wood coatings. J. For. Eng..

[3] Jiang F., Li T., Li Y.J., Zhang Y., Gong A., Dai J.Q., Hitz E., Luo W., Hu L.B. (2018). Wood-based nanotechnologies toward sustainability. Adv. Mater..

[4] Yang Y.Q., Xu W., Liu X. (2021). Study on permeability of cunninghamia lanceolata based on steam treatment and freeze treatment. Wood Res. Slovakia..

[5] Cai Z., Zhu H., Wang P., Wu C., Gao W., Mu J., Wei S. (2020). Performance optimization of UV curable waterborne polyurethane acrylate wood coatings modified by castor oil. J. For. Eng..

[6] Zhang S.W., Yu A.X., Song X.Q., Liu X.Y. (2013). Synthesis and characterization of waterborne UV-curable polyurethane nanocomposites based on the macromonomer surface modification of colloidal silica. Prog. Org. Coat..

[7] Jiang L., Shen J., Zhao Z., Dong H., Li Y. (2019). Study on film properties and VOCs of nano-TiO_2_ and ZnO modified waterborne paints. J. For. Eng..

[8] Scheiner M., Dickens T.J., Okoli O. (2016). Progress towards self-healing polymers for composite structural applications. Polymer.

[9] Lin X., Su J., Feng B., Guo S., Chen Y., Liu P., Wei S. (2019). Modification of waterborne acrylate coatings using biomass silicon. J. For. Eng..

[10] Chen K.L., Zhou J.L., Che X.G., Zhao R.Y., Gao Q. (2020). One-step synthesis of core shell cellulose-silica/n-octadecane microcapsules and their application in waterborne self-healing multiple protective fabric coatings. J. Colloid Interface Sci..

[11] Yan X., Qian X., Wu Z. (2019). Self-repairing technology of microencapsulate and its applications in coatings. J. For. Eng..

[12] Revuelta M.V., Bogdan S., Gamez-Espinosa E., Deya M.C., Romagnoli R. (2020). Green antifungal waterborne coating based on essential oil microcapsules. Prog. Org. Coat..

[13] Busch L., Avlasevich Y., Zwicker P., Thiede G. (2021). Release of the model drug SR101 from polyurethane nanocapsules in porcine hair follicles triggered by LED-derived low dose UVA light. Int. J. Pharm..

[14] Ghorbani M., Ebrahimnezhad-Khaljiri H., Eslami-Farsani R., Vafaeenezhad H. (2021). The synergic effect of microcapsules and titanium nanoparticles on the self-healing and self-lubricating epoxy coatings: A dual smart application. Surf. Interfaces.

[15] Xiao D.X., Liang W.L., Xie Z.G., Cheng J.L., Du Y.J., Zhao J.H. (2020). A temperature-responsive release cellulose-based microcapsule loaded with chlorpyrifos for sustainable pest control. J. Hazard. Mater..

[16] Wang X.G., Zhang C.Y., Wang K., Huang Y.Q., Chen Z.F. (2020). Highly efficient photothermal conversion capric acid phase change microcapsule: Silicon carbide modified melamine urea formaldehyde. J. Colloid Interface Sci..

[17] Tan X.Y., Zhang J.P., Guo D., Sun G.Q., Zhou Y.Y., Zhang W.W., Guan Y.S. (2020). Preparation, characterization and repeated repair ability evaluation of asphalt-based crack sealant containing microencapsulated epoxy resin and curing agent. Constr. Build. Mater..

[18] Wei Y.S., Xiong Y.M., Guo B.M., Yang H.B. (2020). Study on the influencing factors of the emulsion stability of a polymeric surfactant based on a new emulsification device. Energies.

[19] Ma L.L., Shang Y.N., Zhu Y.D., Zhang X.N., Jingjing E., Zhao L.H., Wang J.G. (2020). Study on microencapsulation of lactobacillus plantarum LIP-1 by emulsification method. J. Food Process Eng..

[20] Patel A.S., Kar A., Mohapatra D. (2020). Development of microencapsulated anthocyanin-rich powder using soy protein isolate, jackfruit seed starch and an emulsifier (NBRE-15) as encapsulating materials. Sci. Rep..

[21] Li H.Y., Cui Y.X., Li Z.K., Zhu Y.J., Wang H.Y. (2018). Fabrication of microcapsules containing dual-functional tung oil and properties suitable for self-healing and self-lubricating coatings. Prog. Org. Coat..

[22] Pedaballi S., Li C.C., Song Y.J. (2019). Dispersion of microcapsules for the improved thermochromic performance of smart coatings. RSC Adv..

[23] Zandi M.S., Hasanzadeh M. (2017). The self-healing evaluation of microcapsule based epoxy coatings applied on AA6061 Al alloy in 3.5% NaCl solution. Anti-Corros. Methods Mater..

[24] Yan X.X., Tao Y., Chang Y.J. (2021). Effect of shellac waterborne coating microcapsules on the optical, mechanical and self-healing properties of waterborne primer on *Tilia europaea* L. wood. Coatings.

[25] Durmaz S., Ozgenc O., Avci E., Boyaci I.H. (2020). Weathering performance of waterborne acrylic coating systems on flat-pressed wood-plastic composites. J. Appl. Polym. Sci..

[26] Li J., Shan W.W., Cui J.C., Qiu H.X., Yang G.Z., Zheng S.Y., Yang J.H. (2020). Enhanced corrosion resistance and weathering resistance of waterborne epoxy coatings with polyetheramine-functionalized graphene oxide. J. Coat. Technol. Res..

[27] Wu Y., Wu J.M., Wang S.Q., Feng X.H., Chen H., Tang Q.W., Zhang H.Q. (2019). Measurement of mechanical properties of multilayer waterborne coatings on wood by nanoindentation. Holzforschung.

[28] Meng Y., Deng J.X., Lu Y., Wang S.J., Wu J.X., Sun W. (2021). Fabrication of AlTiN coatings deposited on the ultrasonic rolling textured substrates for improving coatings adhesion strength. Appl. Surf. Sci..

[29] Liu Y., Hu J., Wu Z. (2020). Fabrication of coatings with structural color on a wood surface. Coatings.

[30] Liu Y. (2021). Self-assembly of poly (styrene-methyl methacrylate-acrylic acid) (P(St-MMA-AA)) colloidal microspheres on wood surface by thermal-assisted gravity deposition. Wood Sci. Technol..

[31] Liu Y., Hu J. (2021). Investigation of polystyrene-based microspheres from different copolymers and their structural color coatings on wood surface. Coatings.

